# Photosealed Neurorrhaphy Using Autologous Tissue

**DOI:** 10.3390/ijms25136958

**Published:** 2024-06-25

**Authors:** Nicolò Rossi, Maria Bejar-Chapa, Riccardo Giorgino, Benjamin B. Scott, David M. Kostyra, Giuseppe M. Peretti, Mark A. Randolph, Robert W. Redmond

**Affiliations:** 1 Wellman Center for Photomedicine, Harvard Medical School, Massachusetts General Hospital, Boston, MA 02114, USA; nicolo.rossi@unimi.it (N.R.); riccardogiorgino93@gmail.com (R.G.); benscottmd@gmail.com (B.B.S.); dmk3mchs@gmail.com (D.M.K.); 2 Plastic Surgery Research Laboratory, Department of Surgery, Harvard Medical School, Massachusetts General Hospital, Boston, MA 02114, USA; mkbejar@gmail.com (M.B.-C.); marandolph@mgh.harvard.edu (M.A.R.); 3 Department of Biomedical Sciences for Health, University of Milan, 20122 Milan, Italy; giuseppe.peretti@unimi.it; 4 IRCCS Ospedale Galeazzi Sant’Ambrogio, 20157 Milan, Italy

**Keywords:** rose bengal, neurorrhaphy, sciatic nerve, nerve repair, photochemistry amniotic membrane, crosslinking, microsurgery

## Abstract

Photochemical sealing of a nerve wrap over the repair site isolates and optimizes the regenerating nerve microenvironment. To facilitate clinical adoption of the technology, we investigated photosealed autologous tissue in a rodent sciatic nerve transection and repair model. Rats underwent transection of the sciatic nerve with repair performed in three groups: standard microsurgical neurorrhaphy (SN) and photochemical sealing with a crosslinked human amnion (xHAM) or autologous vein. Functional recovery was assessed at four-week intervals using footprint analysis. Gastrocnemius muscle mass preservation, histology, and nerve histomorphometry were evaluated at 120 days. Nerves treated with a PTB-sealed autologous vein improved functional recovery at 120 days although the comparison between groups was not significantly different (SN: −58.4 +/− 10.9; XHAM: −57.9 +/− 8.7; Vein: −52.4 +/− 17.1). Good muscle mass preservation was observed in all groups, with no statistical differences between groups (SN: 69 +/− 7%; XHAM: 70 +/− 7%; Vein: 70 +/− 7%). Histomorphometry showed good axonal regeneration in all repair techniques. These results demonstrate that peripheral nerve repair using photosealed autologous veins produced regeneration at least equivalent to current gold-standard microsurgery. The use of autologous veins removes costs and foreign body concerns and would be readily available during surgery. This study illustrates a new repair method that could restore normal endoneurial homeostasis with minimal trauma following severe nerve injury.

## 1. Introduction

Despite considerable progress in many aspects of reconstructive microsurgery, peripheral nerve injury still represents a significant clinical challenge. It has been estimated that more than 2 million people suffer peripheral nerve injuries each year, mainly affecting young and healthy patients [1,2,3]. Microsurgical suture repair is the gold standard, but outcomes are often inadequate, even with optimal surgical treatment. Poor functional recovery and common sequelae such as sensory and motor dysfunction and neuroma formation considerably impact quality of life, leading to a severe socioeconomic burden [2,4,5,6,7].

To address this issue, most recent studies have examined biological changes that follow peripheral nerve injury as well as surgical techniques and biomaterials utilized for repair. Although the exact molecular mechanisms are only partially understood, the critical aspects following a peripheral nerve injury are well known. Damaged axons lose their normal intraneural function, and an extensive regeneration process guides and promotes axonal repair [8].

Schwann cells play an essential role in this process since they initiate upregulation and downregulation of a series of genes in an attempt to restore normal intraneural homeostasis [9]. Schwann cells, along with macrophages, participate in the Wallerian degeneration process that consists of initial clearance of damaged axons from the proximal and distal nerve stumps. Subsequently, Schwann cells release neurotrophic factors to support axonal regeneration and regrowth. The goal of an optimal regrowth microenvironment is crucial to recreating nerve continuity [9,10]. Moreover, several studies have underlined the positive effect of vascular endothelial growth factor (VEGF) on Schwann cell proliferation and nerve regeneration with a mix of neurotrophic and neuroprotective effects [11,12].

With conventional suture repair, the ultimate goal of sealing the repair site and isolating it from the surrounding tissues is not always achieved. Without sealing, the concentration of growth factors is locally reduced, which hinders regeneration of axons and restoration of nerve function [13]. Although microsurgical techniques enable approximation of the proximal and distal ends, other drawbacks should be considered. Firstly, the passage of the needle through the epineurium is traumatic and can alter the complex mechanism of nerve regeneration. Secondly, suture material left after nerve repair can increase the chance of inflammation and scar formation with substantial repercussions on axon regeneration [14].

Thus, researchers have pursued alternative repair techniques that could isolate the repair site from the surrounding microenvironment and regenerate homeostasis [15]. Fibrin glue has been tested, with mixed results [16,17,18,19]. Koulaxosidis et al. found that fibrin glue repair led to enhanced axonal elongation during early peripheral nerve regeneration in a mouse model [16]. Conversely, Maragh et al. reported a failure rate of 13% after fibrin glue repair and no failures after standard suture repair in a rat model [17]. The combination of fibrin glue and nerve growth factors or mesenchymal stem cells has also been investigated with promising results, but further analyses are required [18,19]. Our own studies with fibrin glue in a rat sciatic nerve model proved less successful than standard microsurgery or PTB [20].

Photochemical tissue bonding (PTB) employs a photoactivated dye to crosslink a nerve wrap to the epineurial surface resulting in a tight seal around the neurorrhaphy to isolate and optimize the regenerating nerve microenvironment, while reducing the suture burden. We previously demonstrated the ability of PTB to bond and seal several tissues using different materials, including human amniotic membrane (HAM) [21]. This approach has demonstrated a superior regenerative outcome for nerve deficit repair compared to microsurgery and fibrin glue [20].

Amnion is a somewhat immune-privileged tissue with a broad number of beneficial effects. Its commercial availability and ease of manipulation is attractive as a tool in regenerative medicine [22]. Although human amnion has some immune privilege, subsequent commercial processing, cost, and foreign body concerns may limit its use as a wrap in PTB for peripheral nerve repair. In contrast, veins are “free”, often readily available, and can be harvested with minimal additional morbidity to the patient, or even utilized as a spare part from an amputated limb if present. As an autologous tissue harvested from the patient, there are no immunoreactivity concerns that could cause adhesion scarring or secondary compression. We have already shown amnion and veins to be highly effective as nerve photosealants in preventing neuroma formation after peripheral nerve injury in a rat sciatic nerve transection model [23]. While care must be taken in applying nerve wrap materials to ensure compression does not result when sutured in place, both vein and amnion are ideal in terms of their ability to self-adhere to the nerve prior to photosealing without causing compression [24,25].

In this study, we investigated the efficacy of autologous veins as a photosealed wrap over 16 weeks in a sciatic nerve transection and repair model, testing the motor and sensory function of the limb combined with histologic assessment. The photosealed vein was compared to photosealed xHAM as a positive control for photosealing and also to the standard of care microsurgical neurorrhaphy. The overall goal of this study was to identify a peripheral nerve repair technique that could restore normal endoneurial homeostasis in a less traumatic fashion while removing any concerns of immunogenicity of the wrap material. If so, this may ultimately bridge the gap that still exists in the field of nerve reconstructive microsurgery.

## 2. Results

### 2.1. Functional Recovery

Animals treated with an autologous vein wrap and sealed with PTB (experimental group 3) showed better SFI compared to the other groups at day 120 (Table 1). Nevertheless, there was no statistical difference between the sciatic function indices of the autologous vein and xHAM wraps and the group treated with standard neurorrhaphy.

At earlier time points it was difficult to calculate SFI due to inconclusive heel prints due to the animals dragging the denervated foot in the walking tracks. However, the toe prints were clear in all cases allowing the measurement of toe spreads. Overall, when measuring outer toe spread, the group with the autologous vein wrap sealed with PTB showed a slight improvement at each timepoint from day 30 to day 120 (Table 2), without reaching statistical significance.

### 2.2. Gastrocnemius Muscle Mass Retention

Percentage gastrocnemius muscle mass retention is shown in Table 1 and was very similar in groups with no statistically significant differences.

### 2.3. Histology and Histomorphometry

**Figure 1:**
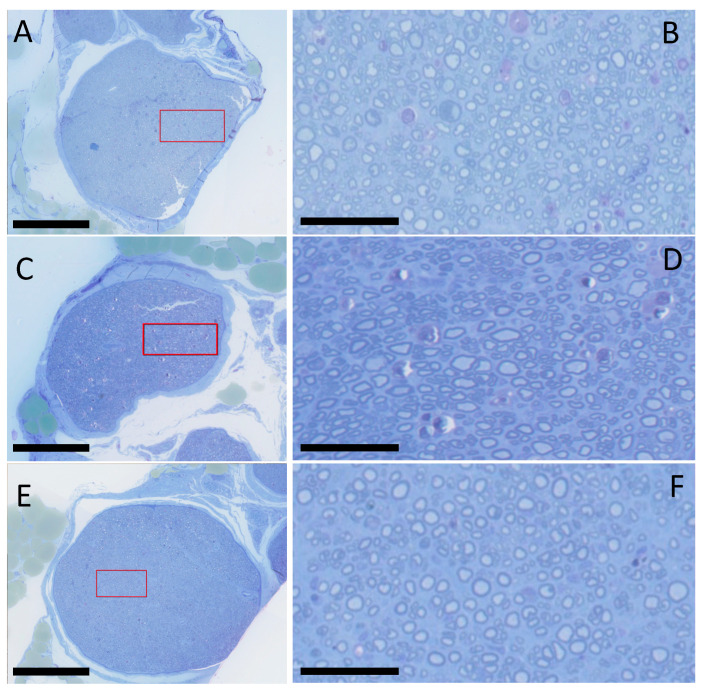
Toluidine blue-stained cross-sections of nerve 5 mm distal to the repair site showing myelinated axons. (**A**) Standard neurorrhaphy. (**B**) Magnified inset (red box) of standard neurorrhaphy group. (**C**) Photosealed xHAM. (**D**) Magnified inset (red box) of photosealed xHAM. (**E**) Photosealed autologous vein. (**F**) Magnified inset (red box) of photosealed autologous vein. Scale bars for (**A**,**C**,**E**) = 250 µm. Scale bars for (**B**,**D**,**F**) = 50 µm.

Toluidine blue-stained sections showed the presence of regenerated myelinated axons both proximal and distal to the repair site in all of the experimental groups (Figure 1). Table 1 details the histomorphometric analysis for the distal to proximal (D/P) intrafascicular area ratio and D/P axon diameter ratio for each group. The intrafascicular area ratio (D/P) was found to be higher in the photosealed groups (PTB/Vein: 0.95 +/− 0.19; PTB/xHAM: 0.98 +/− 0.19) than the microsurgery group (SN: 0.84 +/− 0.22), with high values indicative of excellent axonal regeneration and intraneural organization. Axon diameters are significantly smaller in distal sections in all groups, but the ratio (D/P) showed no statistically significant difference between groups (PTB/Vein: 0.80 +/− 0.26; PTB/xHAM: 0.78 +/− 0.20; SN: 0.77 +/− 0.17).

## 3. Discussion

In this study, we compared regenerative outcomes following photosealing with a vein to microsurgery (a standard of care reference) and photosealing with human amniotic membrane, which was previously demonstrated in our group to produce excellent regenerative outcomes in both transection and nerve deficit repair [22,26,27,28,29,30]. Results demonstrate that photochemical sealing of the peripheral nerve repair site with an autologous vein can result in functional and histological regeneration similar to standard microsurgical neurorrhaphy and is equivalent to photosealing with human amniotic membrane. Amnion is a thin (~30 μm) membrane that can be easily wrapped around the peripheral nerve repair site, and its transparency allows the activating green light to penetrate through the amnion to the epineural–amnion interface [31,32] for efficient crosslinking. Additionally, amnion contains many growth factors that assist in wound healing and tissue regeneration [33] and has been used in a wide range of tissue repair models [34,35,36,37,38,39]. Many commercial nerve wraps do not possess the above characteristics that are essential for a photosealed wrap.

However, commercial amniotic membrane products, derived from human placenta, must undergo strict screening for potential infectious diseases and post-processing may also contribute to immunoreactivity, despite all efforts to minimize foreign body reactions. These products are also costly, and the combination of high cost and potential immunoreactivity has led us to investigate alternatives that retain the efficacy of photosealed HAM in nerve repair. While veins are slightly thicker and not quite as translucent as HAM, they allow sufficient light penetration through the vein to the vein–epineurium interface to activate crosslinking to seal the repair site. Rose bengal does not penetrate into the bulky vein, so reactivity is limited to the interfacial side of the vein in contact with the epineurium [40,41]. Crosslinking is evident in the PTB/vein group through photobleaching of the red color of RB, similar to that seen for PTB/xHAM.

Interestingly, the positive outcomes of the human amniotic membrane + PTB and autologous vein + PTB highlight the broad applicability of photochemical technology with biological wraps. The beneficial effect of biological wraps on functional outcomes and nerve regeneration relied on the photochemical sealing process. It has already been demonstrated that the collagen content is mandatory when PTB is applied, and that rose bengal creates covalent crosslinks between collagen molecules on tissue surfaces [21]. Our studies showed that the amnion and epineurium stromal layer comprises collagen and can seal in a watertight fashion [27,30,42]. We postulate that the endothelial surface is also rich in collagen, thin, and translucent, suggesting that photochemical tissue bonding occurs to seal off the endoneurium from the outside environment. Even if not reaching statistical significance, the autologous vein wrap + PTB method showed slightly better results than the amnion wrap, suggesting that sealing the autologous vein wrap with PTB over the repair site is a viable approach.

Excellent outcomes of the autologous vein group in terms of intrafascicular area ratio (D/P), axon diameter ratio (D/P), and sciatic function index may be linked to early restoration of the blood–nerve barrier function, which is a significant factor in creating a suitable microenvironment for axonal regeneration [43]. After nerve transection injury, the blood–nerve barrier becomes more permeable, allowing proteins to enter the endoneurium. Although the exact role of the blood–nerve barrier in aiding neural repair is not clear, the necessity of restoring endoneurial homeostasis has already been established [44].

This study tested the ability of two clinically available materials (HAM and autologous veins) to photoseal a peripheral nerve repair site to optimize regeneration. Both photosealed wraps led to good functional and histological outcomes. PTB of nerve repairs with biological membranes has additional benefits such as reducing the number of epineurial sutures, decreasing trauma and axonal escape, and reduced foreign body responses such as fibrosis and inflammation with minimal additional morbidity for patients [45,46]. Other advantages of this technique are reduced operative time and ease of surgical technique to accomplish nerve transection repairs when compared to microsurgery. An additional advantage specific to photosealing with veins is that autologous veins are often readily available at the time of injury and can be easily collected during surgery with little adverse effect on the patient, nor additional financial cost.

In photosealing, the ability to form an intimate seal over the repair site is crucial to optimize the regenerative endoneurial environment. Photosealing is essentially applicable to all collagenous materials with sufficient pliability and transparency to act as nerve wraps. Newer nerve wrap initiatives include a drug delivery aspect to enhance regeneration (e.g., tacrolimus [47]). The crosslinking process modifies the tissue to a degree and we have seen inhibition of inflammatory cell migration in some tissues following the process [48]. However, it is very unlikely that small molecular and even macromolecular drugs would be inhibited from diffusing to target tissue. Thus, we believe that photosealing would also be applicable to such materials.

In peripheral nerve repair, the only FDA-approved materials are for nerve conduit. These conduits are made of different biomaterials such as type I collagen, polyglycolic acid (PGA), poly-DL-lactide-co-caprolactone (PLCL), and polyvinyl alcohol (PVA), and they can be bioresorbable, such as Neurotube (PGA), Neurolac (PLCL), NeuraGen (type I collagen), and NeuroMatrixNeuroflex (type I collagen), or non-resorbable, such as SaluBridge (PVA hydrogel) [49,50]. Nevertheless, results are poor mainly due to the thickness of the materials and manipulation difficulties [51,52]. Autologous tissue would remove issues related to regulatory authorization with no need for FDA approval.

## 4. Materials and Methods

### 4.1. Preparation of Crosslinked Human Amnion (xHAM)

Placenta obtained from healthy donors following cesarian delivery was stripped of chorion, yielding HAM. The procedure for preparation of crosslinked HAM (xHAM) was performed as previously described [23]. The xHAM was washed 5 times with sterile PBS to ensure no crosslinking solution remained and was then placed on sterile parafilm in PBS before use.

### 4.2. Surgical Procedures

The Massachusetts General Hospital Institutional Animal Care and Use Committee (IACUC) and the Animal Care and Use in Research Office (ACURO) of the US Army Medical Research and Development Command approved all procedures in this study. Furthermore, all experiments were conducted and reported in accordance with the ARRIVE guidelines [53]. Forty-two female Lewis rats weighing 220–250 g were purchased from Charles River Laboratories (Wilmington, MA, USA) and were acclimatized for three days before surgery in standard animal facilities at the Massachusetts General Hospital. Thirty-six animals were randomized in three groups (standard neurorrhaphy, photosealed xHAM wrap, and photosealed autologous vein wrap) and six animals were used as bilateral femoral vein donors. The animals received pre-operative doses of Buprenorphine (0.01 mg/kg s.c.) and Carprofen (5 mg/kg s.c.) and were anesthetized with Isoflurane inhalation (1–5%), titrated to effect. The fur of the right hindlimb on each animal was then shaved and the skin prepared with betadine. A dorsolateral, muscle-splitting 2 cm incision was made over the right hindquarter of each animal with a No. 15 scalpel. The sciatic nerve was exposed and isolated from the sciatic notch to the distal trifurcation. Under an operating microscope, the nerve was then sharply transected approximately 1 cm proximal to the distal trifurcation. Rats were then randomized to one of three experimental groups, listed below.

Group 1: Standard Neurorraphy (n = 12)

This group of animals received standard microsurgical neurorrhaphy. The proximal stump was reconnected to the distal nerve stump with 10-0 nylon epineurial sutures, approximately 6 sutures depending on nerve size. No photochemical tissue bonding was performed;

Group 2: Photosealed xHAM Wrap (n = 12).

This group received xHAM wrap, sealed over the neurorraphy using PTB. The proximal stump was reconnected to the distal nerve stump with only 2 epineurial sutures (180 degrees apart) with 10-0 nylon. A sterile solution of 0.1% *w*/*v* rose bengal (RB) (4,5,6,7-tetrachloro-2’,4’,5’,7’-tetraiodofluorescein) was prepared by dissolving RB (Sigma-Aldrich) in sterile PBS (Sigma-Aldrich, St. Louis, MO, USA)) and filtering through a 0.22 µm syringe filter (EMD Millipore, Burlington, MA, USA). The human amnion was trimmed to a 1 × 1 cm section, gently dried with a cotton swab, and RB solution was applied to the surface to contact the nerve. The wrap was shielded from light and the dye was allowed to infiltrate the wrap material for a period of 4 min and excess dye was removed by blotting with a sterile cotton swab. The RB-impregnated xHAM was then carefully wound around the neurorraphy site using microforceps, ensuring very close contact between the stained portion of the wrap and the epineurium. The wrap material was bonded to the epineurium via illumination at 532 nm from a continuous-wave KTP laser (Laserscope Aura-i, San Jose, CA, USA; irradiance of 0.4 W/cm^2^) in two increments of 180 degrees around the nerve stump for 60 s each. Photobleaching of RB dye observed postillumination indicated that photosealing reactions had occurred;

Group 3: Photosealed Autologous vein Wrap (n = 12).

This group of animals received an autologous vein wrap sealed over the neurorraphy using PTB. Six donor Lewis rats were anesthetized, and the fur of their bilateral inguinal regions were shaved and the skin prepared with betadine. Bilaterally, a 2 cm incision was made in the groin crease with a No.15 blade. Dissection was performed to expose the femoral vessels under an operating microscope. Following ligation of branches, the femoral veins were sharply excised. The vein grafts were copiously irrigated with heparinized saline and then stored in PBS prior to immediate use. The sciatic nerve was then exposed and transected in the recipient animal. Neurorrhaphy was performed with 2 epineurial sutures 180 degrees apart with 10-0 nylon to reconnect the proximal stump and distal nerve stumps. A sterile solution of 0.1% of RB (*w*/*v*) was applied to the endothelial surface, and the dye was allowed to absorb for 4 min. Once approximated, the RB-stained femoral vein wrap was applied and then exposed to green light using the parameters described for Group 2.

**Figure 2:**
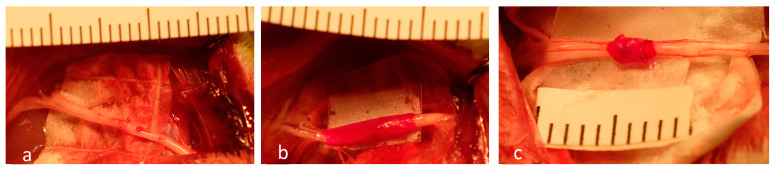
Photographs of immediate post-repair sciatic nerves in all treatment groups. (**a**): standard neurorrhaphy; (**b**): xHAM + PTB; and (**c**): vein + PTB.

In all groups, the surgical wounds were subsequently closed in multiple layers with absorbable suture. All animals had subsequent free access to food and water. Figure 2 shows examples of immediate post-repair nerves in all three groups.

### 4.3. Functional Recovery

**Figure 3:**
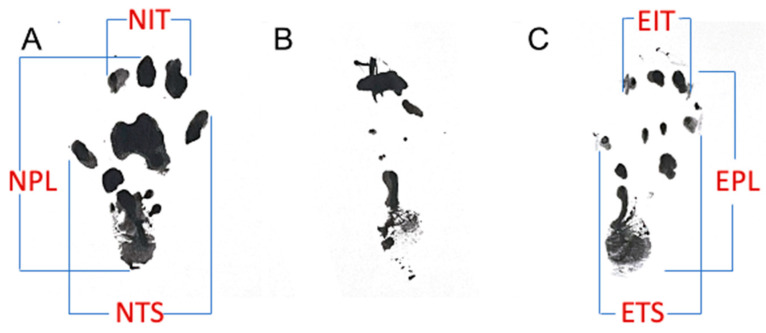
Representative rodent footprints and analysis. (**A**) Pre-operative (normal). (**B**) Immediately post-op (no recovery). (**C**) 16 weeks post-op (partial recovery) for photosealed vein, showing measurement parameters and equation for sciatic function index (SFI) calculation. NPL and EPL (normal and experimental print length; NTS and ETS (normal and experimental toe spread); NIT and EIT (normal and experimental intermediate toe spread).

Walking tracks for all animals were performed 1 day prior to the initial surgery, one day prior to euthanasia (120 days), and at intermediate timepoints of 30, 60, and 90 days postoperatively. Rats had their hind paws dipped in ink and traversed a walking track with their footprints recorded on paper. SFI measurements were obtained from footprints using Equation (1), as described by Sarikcioglu et al. [54] (Figure 3).

(1) $$\mathrm{SFI}=-38.3\left(\frac{\mathrm{EPL}-\mathrm{NPL}}{\mathrm{NPL}}\right)+109.5\left(\frac{\mathrm{ETS}-\mathrm{NTS}}{\mathrm{NTS}}\right)+13.3\left(\frac{\mathrm{EIT}-\mathrm{NIT}}{\mathrm{NIT}}\right)-8.8$$

The subsequent SFI values from day 120 were compared to day 30 measurements, as previously described [55]. Additionally, outer toe spread was measured at each time point.

### 4.4. Gastrocnemius Muscle Mass Retention

Animals were euthanized postoperatively at day 120 via injection of pentobarbital (100 mg/kg, i.p.) (Euthasol, Virbasc AH, Inc., Fort Worth, TX, USA). The right (operative) and left (control) gastrocnemius muscles were carefully dissected under the operative microscope. The muscles were weighed, and their wet weights compared to analyze the percentage of gastrocnemius muscle mass preserved in the experimental leg.

### 4.5. Histology and Histomorphometry

Samples were prepared for histology as previously described [20]. Tissues were fixed in Karnovsky fixative (K2 buffer) (Electron Microscopy Sciences, EMS, Hatfield, PA, USA), 2.5% glutaraldehyde (EMS), and 2% paraformaldehyde in 0.1 M sodium cacodylate buffer (wash buffer) (EMS) at 4 °C for up to 24 h. After washing, tissues were post-fixed with 1% OsO_4_ (EMS) plus 1.5% Potassium Ferrocyanide (EMS) in a wash buffer, for 1.5 h at 4 °C. After washing, the tissues were treated with 1% Tannic Acid (EMS) in ddH^2^O for 40 min, and were then en bloc stained with 1% uranyl acetate substitute (EMS) for 20 min and 3% lead Citrate (EMS) for 15 min. Tissues were then dehydrated in gradient alcohol series (50%, 70%, 95%, and 100%), infiltrated with propylene oxide/Epon t812 gradient mix (100%/0%, 70%/30%, 50%/50%, 30%/70%, and 0%/100%), and embedded in Epon t812 (Tousimis, Rockville, MD, USA). Semi-thin cross sections (0.5 µm) were cut and stained with toluidine blue. Histologic specimens were digitally imaged with a Hamamatsu NanoZoomer 2.0-HT slide scanner (Meyer Instruments, Houston, TX, USA) and then evaluated using NDP2 software (Hamamatsu Corp., Bridgewater, NJ, USA). Images from sections taken 5 mm distal to the neurorraphy were analyzed.

Histomorphometric scoring of peripheral nerve regeneration can be undertaken in various ways. Factors such as axon diameter and count, myelin thickness, and the G-factor have all been used to analyze regenerative outcomes [28,29]. Following our experience in these previous studies, we addressed nerve histomorphometry via a different approach. All areas in the nerve cross-section that encompass distinct axon-containing nerve fascicles were summed for each image. Additionally, average axon diameter was also measured from axons contained withing distinct fascicles. A minimum of 3 sections per specimen were imaged and analyzed using NDP2 software (Hamamatsu Corp., Bridgewater, NJ, USA) and evaluated by three independent evaluators, blinded to the treatment/control nature of the samples. Evaluators choose the fascicular areas and individual axons for axon diameter measurement. Sections were taken ~5 mm proximal (P) and distal (D) to the neurorraphy, reflecting normal and regenerated nerve architecture, respectively. The mean of the three measurements in each case is presented and excellent agreement was observed between evaluators’ analyses in all analyses.

### 4.6. Statistical Analysis

Statistical analysis was performed using KaleidaGraph for Windows v4.1 (Synergy Software, Reading, PA, USA). Sciatic Function Index (SFI) and outer toe spread data were analyzed using repeated measures analysis of variance to detect the existence of significant differences over time. All remaining analysis between treatment groups was performed using ANOVA and the post hoc Bonferroni test. Values of *p* < 0.05 were considered statistically significant.

## 5. Conclusions

The results of this study demonstrated that photosealing the peripheral nerve repair site with an autologous vein wrap is effective in terms of functional recovery and histomorphometric regeneration parameters, compared to conventional suture neurorrhaphy. The effectiveness of the PTB/vein method could facilitate the clinical applicability of PTB, avoiding costs and foreign body concerns of amnion-derived products or other natural or synthetic wrap materials.

## Figures and Tables

**Table 1:** Regenerative outcome metrics at end of study (120 days). Values are provided as mean +/− standard deviation for n = 8–12 per group.

Group	SFI (120 Days)	% Muscle Mass	Intrafascicular Area Ratio (D/P)	Axon Diameter Ratio (D/P)
Microsurgery	−58.4 +/− 10.9	69 +/− 7	0.84 +/− 0.22	0.77 +/− 0.17
PTB/xHAM	−57.9 +/− 8.7	70 +/− 7	0.98 +/− 0.19	0.78 +/− 0.20
PTB/vein	−52.4 +/− 17.1	70 +/− 7	0.95 +/− 0.19	0.80 +/− 0.26

**Table 2:** Experimental toe spread (ETS) measurements in mm at each timepoint. Values are provided as mean +/− standard deviation for n = 12 per group.

Group	Preop	30 Days	60 Days	90 Days	120 Days
Microsurgery	19.4 +/− 1.8	7.9 +/− 2.2	8.5 +/− 2.7	12.8 +/− 2.3	11.7 +/− 1.9
PTB/xHAM	19.9 +/− 0.9	8.5 +/− 1.6	10.0 +/− 3.5	12.0 +/− 1.6	12.1 +/− 1.3
PTB/vein	19.2 +/− 0.7	8.7 +/− 1.9	11.2 +/− 1.8	12.7 +/− 2.1	12.9 +/− 2/3

## Data Availability

The raw data supporting the conclusions of this article will be made available by the authors on request.
